# Transcriptional state dynamics lead to heterogeneity and adaptive tumor evolution in urothelial bladder carcinoma

**DOI:** 10.1038/s42003-023-05668-3

**Published:** 2023-12-21

**Authors:** Antara Biswas, Sarthak Sahoo, Gregory M. Riedlinger, Saum Ghodoussipour, Mohit K. Jolly, Subhajyoti De

**Affiliations:** 1 Rutgers Cancer Institute of New Jersey, Rutgers the State University of New Jersey, New Brunswick, NJ USA; 2 Indian Institute of Science, Bangalore, India

**Keywords:** Molecular medicine, Gene regulatory networks

## Abstract

Intra-tumor heterogeneity contributes to treatment failure and poor survival in urothelial bladder carcinoma (UBC). Analyzing transcriptome from a UBC cohort, we report that intra-tumor transcriptomic heterogeneity indicates co-existence of tumor cells in epithelial and mesenchymal-like transcriptional states and bi-directional transition between them occurs within and between tumor subclones. We model spontaneous and reversible transition between these partially heritable states in cell lines and characterize their population dynamics. SMAD3, KLF4 and PPARG emerge as key regulatory markers of the transcriptional dynamics. Nutrient limitation, as in the core of large tumors, and radiation treatment perturb the dynamics, initially selecting for a transiently resistant phenotype and then reconstituting heterogeneity and growth potential, driving adaptive evolution. Dominance of transcriptional states with low *PPARG* expression indicates an aggressive phenotype in UBC patients. We propose that phenotypic plasticity and dynamic, non-genetic intra-tumor heterogeneity modulate both the trajectory of disease progression and adaptive treatment response in UBC.

## Abstract (web summary)

A set of functional genomics and statistical analyses identifies and establishes key determinants of transcriptional state dynamics and plasticity during tumor growth and under treatment in urothelial bladder carcinoma.

## Introduction

Urothelial bladder carcinoma (UBC) is a major cause of morbidity and mortality among adults with over 500,000 new cases and 200,000 deaths annually worldwide^1,2^. Due to the costs of treatment and intensive surveillance required, UBC has the highest lifetime treatment costs per patient from diagnosis to death of all cancers^3^. Treatment efficacy, especially for muscle invasive and metastatic urothelial cancer, is limited because the tumors tend to be heterogeneous, which contributes to poor risk stratification, emergence of resistance, and ultimately poor clinical outcome^4–6^.

While genetic heterogeneity in UBC is well documented^4,6,7^, single cell analysis has indicated extensive transcriptomic heterogeneity in tumor cell populations in UBC ^8–10^, which have led to debates whether intra-tumor transcriptomic heterogeneity is a surrogate of genetic variation between subclones, gene expression noise, or indicate functional variations around phenotypically distinct transcriptional states independent of genetic heterogeneity^11–13^. Intra-tumor transcriptomic state heterogeneity can benefit tumor adaptation by conferring phenotypic plasticity—characterized by epigenetic remodeling in tumor cells and their ability to acquire reversible characteristics such as EMT, multilineage differentiation potential and self-renewal, that enable them to cope with stresses during tumor progression, metastatic cascade or therapy^14–17^. Evidence from multiple cancer types^17–19^ suggest that, while the above factors likely contribute to transcriptomic heterogeneity, co-existence of multiple cell states may be pervasive^20–23^, providing evolutionary advantage to neoplasms via increased immune evasion, drug resistance, and invasiveness^24,25^. It is unclear whether an equivalent transcriptional state dynamics also contributes to clinically relevant functional intra-tumor heterogeneity in UBC, and if a minimal regulatory program governing this transcriptional state dynamics can be identified.

**Fig. 1:**
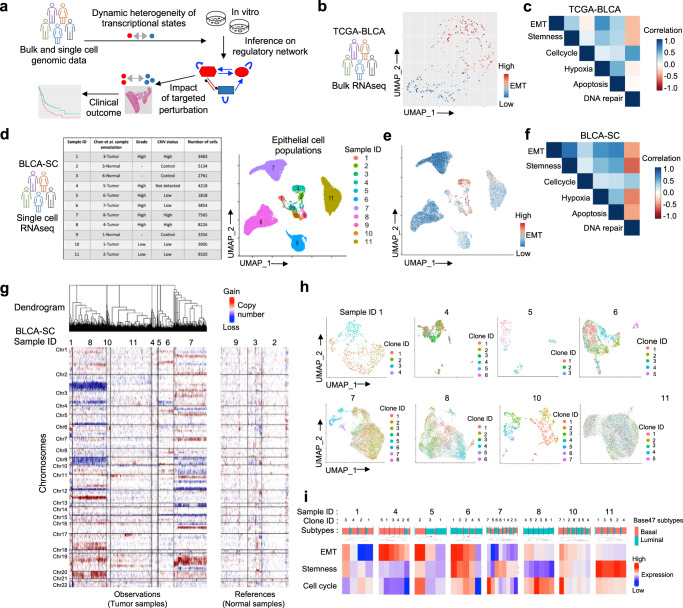
Inter- and intra-tumoral heterogeneity in urothelial bladder cancer patients. **a** A schematic representation of integrative analyses of cell state dynamics in vitro and in vivo in urothelial bladder cancer. **b** UMAP projection of TCGA-BLCA samples (*n* = 408) show inter-tumor heterogeneity in oncogenic pathways, with EMT being a major determinant. Each dot corresponds to a single sample and the color gradient is proportional to the expression of the EMT gene signature. **c** Gene expression correlations between oncogenic signatures in the tumors in the TCGA-BLCA cohort. **d** Summary of all single cell RNAseq (BLCA-SC) samples and UMAP plot of epithelial cells colored by sample ID. Each dot represents a single cell. **e** UMAP plot of epithelial cells colored according to the expression of the EMT gene signature. **f** Gene expression correlations between oncogenic signatures at single cell resolution in the BLCA-SC cohort. **g** RNA expression-guided copy number estimation and inference on clonal architecture in the tumors from the BLCA-SC cohort. Each column corresponds to a cell, ordered by sample and clustered within each sample by chromosomal alteration status. Normal tissue samples are shown to the right. **h** UMAP plots of single cells in transcriptomic space colored by tumor clone IDs derived from chromosomal alteration status. **i** Proportions of cells in basal and luminal subtypes according to the Base47 subtype signature is shown for each clone ID in each tumor in the BLCA-SC cohort. Heatmap shows expression levels of selected oncogenic pathways in tumor clones, as annotated by their IDs in **h**.

In this study, we use single cell genomics data from patient-derived tumors and in vitro evolution in cell line models with targeted perturbations to deconstruct the complexity of dynamic transcriptomic heterogeneity in UBC (Fig. 1a). Our results highlight remarkable plasticity and dynamics of transcriptional states and identify a core regulatory network. We further examine the role of nutrient limitation in tumor microenvironment and radiation treatment on transcriptional state dynamics and assess the potential for targeting sources of variations in intra-tumor heterogeneity and modulating overall cell population-level growth dynamics during tumor progression to improve effective treatment response.

## Results

### Transcriptional state heterogeneity within and between tumors

To assess the extent to which patient-to-patient variation in transcriptomic makeup could be due to heterogeneity in cancer-associated cellular processes we first examined the bulk transcriptomic profiles of 408 muscle invasive and/or metastatic urothelial bladder carcinoma samples from the TCGA (TCGA-BLCA)^26–28^. Single sample gene-set enrichment (ssGSEA) analyses identified an aggressive epithelial mesenchymal transition (EMT) signature as a leading differentiating feature between patient samples in UMAP projections (Fig. 1a–c and Supplementary Fig. 1a, Supplementary Data 2). To interpret the observations considering the major BLCA subtypes, we further classified the tumors into luminal and basal subtypes using the transcriptome Base47 classifier^29^ and found that the tumors with predominantly basal signature had intermediate-to-high EMT scores, while the predominantly luminal tumors had low-to-intermediate score (Supplementary Fig. 1a). This suggests that the transcriptomes of UBC tumors typically had admixtures of both characteristics, irrespective of their subtypes, but some were predominantly either epithelial- or mesenchymal-type. Since bulk RNAseq data carries aggregated transcriptional signals from tumor and non-tumor cells, and different tumor cell populations, limiting our ability to identify biological variations around multiple transcriptomic states, we next examined the transcriptional landscapes and associated contextual molecular signatures at single-cell resolution. First, we analyzed single cell RNAseq (scRNAseq) data of 52,721 single cells from 8 UBC tumor samples and 3 para tumor samples from a published study^10^ (herein BLCA-SC dataset) and used gene expression to distinguish the tumor cells from immune, stromal, and other cell types (Supplementary Fig. 1b). Initial pan-transcriptome analysis revealed that transcriptional states of tumor cells primarily clustered by sample ID, suggesting that patient-specific differences dominated the between-patient transcriptomic heterogeneity. This is likely due to extensive genomic differences between tumors (Fig. 1d, Supplementary Fig. 1b), as reported for other cancer-types^11,19,30^, overshadowing variations in transcriptional programs associated with oncogenic cellular processes.

### Convergent epithelial and mesenchymal like transcriptional states across tumor subclones

Next, we calculated single sample GSEA^31^ scores for cancer hallmark pathways for the tumor cell populations in this cohort at single cell resolution, and found that in most patients, the tumor cells formed multiple comparable clusters based on these signatures (Fig. 1e, f, Supplementary Fig. 1c, d, Supplementary Data 2). While some differences among the transcriptional clusters were patient-specific, other patterns of cluster-wise differences were shared across patients, suggesting the presence of potential, common oncogenic transcriptional states that are utilized by the UBC cells; differences between those states were attributed to variations in stemness, EMT, and proliferation-related genes. These observations suggest, the cell clusters likely represent transcriptional states with coherent and differentially expressed oncogenic signatures, and that the observed transcriptional states are likely proxies of tumor cell phenotypes with different, complex malignant characteristics^12^. We next sought to determine if the observed transcriptional state clusters could be predominantly due to genetic differences between clones, or represent recurrent utilization of tumor cell states, at least partially independent of clonal genetic alterations. We used single cell RNA sequencing-guided copy number inference to reconstruct the clonal architecture for each tumor and identify large copy number variations (CNV) between subclones (Fig. 1g). Inter- and intra-tumoral heterogeneity in the single-cell CNV patterns and clonal architectures were substantial. Nonetheless, overlaying clonal architecture and transcriptional state data, we found that the transcriptional clusters, in most cases, did not segregate by subclonal clusters and subtypes (Fig. 1h). Instead, for each patient, the single-cell expression profiles spanned several clusters with distinct expression patterns discovered by unsupervised clustering showing increasing cellular phenotypic heterogeneity (Fig. 1i). These observations suggest that in most patients, tumor cells from the same subclones were members of multiple distinct transcriptional states, and conversely, cells with different subclones converged to the same transcriptional states. This subclone-transcriptional state promiscuity was observed at any level of tumor phylogeny, suggesting that transition between different transcriptional states likely occurs along most phylogenetic lineages in tumors. However, temporal clonal dynamics involving cell state transition in vivo cannot not be tracked for clinical samples, which only offer a snapshot of pseudo-subclonal architecture that misses out on intermediate states. Furthermore, understanding subclonal composition and predicting the innumerable interactions and interdependencies of cancer subclones with each other and the microenvironment is hindered by the rarity of these events within the primary tumor lesion biopsied for sequencing. This prompted us to examine dynamic properties of transcriptional states, and underlying regulation in laboratory based in vitro evolution using the cell line models.

### Spontaneous cell state transition in in-vitro models

**Fig. 2:**
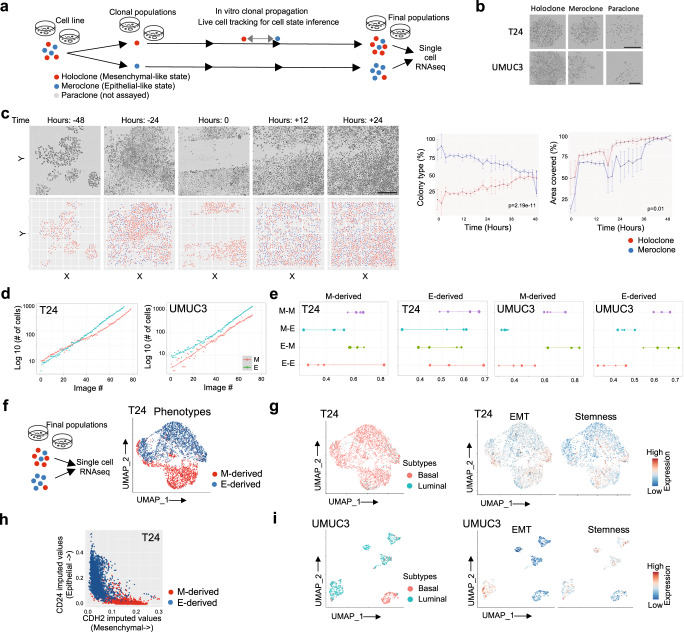
Exploration of cell states in vitro. **a** A workflow illustrating the use of cell line model system to reveal transcriptional state changes by single-cell level phenotypic analysis of large imaging datasets by live-cell tracking and image cytometry. **b** Representative images of holo-, mero- and paraclones 5 days following plating showing characteristic morphologies in indicated bladder cancer cell lines. Scale bars represent 300 μm. **c** LHS : Representative bright-field images and corresponding scatter plots post Cell profiler image segmentation, color annotated by phenotypic classes showing T24 cells forming monolayer and in vitro wound created by straight line scratch across the monolayer and cells’ migration to the wound region with time after initial scratch (0) and every 2 hours post-scratch. RHS : T24 cells wound closure expressed as the cells’ occupancy in wound area and area covered by the cells over time points analyzed with colors indicating phenotypic classes. The results are expressed as mean ± SD. Scale bar represent 300 μm. **d** Plots of the number of T24 and UMUC3 cells in colony from each cell state per image with a smoothed regression line. **e** Dotplot showing T24 and UMUC3 cell state transition probability rates of population to and from each state with colors purple, cyan, green and red indicating cell state transition M-M, M-E, E-M, E-E wherein E and M are epithelial-like and is mesenchymal-like phenotypic classes respectively identified using image dataset, and size of dots indicates grid sizes in images. **f** UMAP analysis of scRNAseq data of T24 cells showing main clusters colored by cell phenotypes. **g** & **i** UMAP analysis of scRNAseq data of T24 and UMUC3 cells showing main clusters colored by Base47 tumor subtypes and signatures where each dot corresponds to a single cell. **h** Scatter plot showing expression of epithelial and mesenchymal marker genes in the annotated cell populations. Imputation was used to avoid zero-inflated representation.

To model key aspects of transcriptional state heterogeneity and heritability, and study dynamics of transition between the transcriptional states, we used two bladder cancer cell lines—T24 and UMUC3 for laboratory based in vitro evolution (Fig. 2a). These cell lines have differential morphology and growth potential—but they spontaneously transition between predominantly three distinct phenotypic states—namely holoclones, meroclones and paraclones (Fig. 2b). Holoclones were clusters of small and tightly packed cells with smooth and defined colony borderlines. Paraclones comprised of dispersed larger cells with undefined border, while meroclones exhibited an intermediate morphology containing a dichotomy of cell shapes and sizes with limited proliferative capacity and irregular boundary. Holoclones are at the apex of cellular hierarchy and are characterized by extensive proliferation and cell renewal capacities, paraclones featured minimal proliferative potential, whereas meroclones have intermediate properties^32–34^ (Supplementary Fig. 2a). From mixed ancestral population of T24 cell line 18% of clones were classified as holoclones, 44% as meroclones and 38% as paraclones. Similarly, for UMUC3, 27%, 41%, 32% were classified as holo-, mero-, and paraclones, respectively (Supplementary Fig. 2a). When plated as single-cells, cells displayed different abilities to spontaneously generate morphologically heterogeneous colonies. Holoclones and meroclones could be re-plated to generate new mixed colonies (Supplementary Fig. 2b). However, paraclones colonies were lost during cultivation for all the three cell lines tested, whereas all the holoclones remained viable (Supplementary Fig. 2c, *p* < 0.0001, Log-rank Mantel-Cox test). Microscopic examination of the seeded wells showed that failure of paraclone cells to form colonies was due to aborted microcolonies that had undergone a few divisions. The cell lines allowed examining cell state dynamics in vitro, and also their underlying transcriptional makeups and regulatory networks.

### Heritability and transition dynamics of cell states during in-vitro evolution

We used a high-definition time-lapse microscopy system and live-cell imaging to track the dynamics of state transition during in vitro evolution in laboratory condition. We tracked T24 and UMUC3 cells over at least 2-3 weeks of culture. In the largest colonies it was only feasible to track cells over 4-5 sequential rounds of cell division so multiple sets of subclones spanning four cellular generations were tracked within each colony. We obtained profiles of single cells from time-lapsed images, extracted quantitative estimates of phenotypic descriptors and used those to annotate populations of cells in the three groups, as above (Supplementary Fig. 2d). We observed that cellular morphologies associated with the phenotypic states were, at least partially, heritable during short-term culturing. Given the differences observed in transcriptional states in different phenotypes as well as the evidence for greater proliferative properties of holoclones, we integrated microscopy approach with functional assay and assessed cell migration in T24 cells by conducting wound healing experiments (Fig. 2c). We found a significant increase in the number of cells that migrated into the wound from the holoclone colonies compared to the meroclone colonies (*p* = 0.01, Wilcox test). At the 12 hours (h) time point, the recovery of the denuded area was recorded as ~67% and 78%, for meroclone and holoclone colonies respectively. The percentage of wound closure at 24 h after wounding was about 75% in meroclone colonies, whereas in holoclone colonies, the percentage was more than 90%. We also observed a significant difference in cell count for both phenotypes in the original scratch over time (*p* = 2.19e-11, Wilcox test). For individual cells identified into holo- and meroclone type, we observed an increase in holoclone cells and corresponding decrease in meroclone cells. A potential mechanism concerns the possible plasticity of meroclone cells reverting to stem-cell-like state and participating in collective migration. These observations show that holoclone cells contribute to wound healing after scratch initiation, and that cell state dynamics are associated with tumor cell population reconstitution and expansion. Furthermore, since holoclone and meroclone colonies consisted predominantly of cells with mesenchymal- (M) and epithelial-like (E) characteristics, the colonies of M and E traits are referred herein as M and E colonies respectively^35,36^. In the light of evidence of transition between mesenchymal and epithelial-like phenotypic states during in vitro evolution, we then calculated state transition rates of T24 and UMUC3 cell lines from live cell imaging data of monoclonally-derived phenotypically distinct colonies. We divided the cell culture plate into grids, classifying the cells with M and E phenotypic states in respective grids, and estimated their relative proportions at a given time (Fig. 2d, Supplementary Fig. 2e). We hypothesized that the cell state transition can be modeled as a continuous time Markov chain process, and accordingly compared between consecutive time-points to determine homo- and heterologous transition rates between states (i.e. mesenchymal-mesenchymal, M-M; mesenchymal-epithelial, M-E; epithelial-mesenchymal, E-M; and epithelial-epithelial E-E; Fig. 2e).We found that in clonally derived cell populations the phenotypic state of cell is retained initially, but within 48 h of plating, the cells start exhibiting phenotypic plasticity and undergo state transitions. In the T24 cell line, the cells derived from M clones showed similar mesenchymal-mesenchymal (M-M, 0.6208 ± 0.04) and epithelial-mesenchymal (E-M, 0.5978 ± 0.05) transition rates for all grid sizes but the lowest rates for mesenchymal-epithelial transitions (M-E,0.4126 ± 0.11) – indicating that cells with M initial state give rise to more mesenchymal like cells as compared to all other states. On the other hand, in the cells derived from E clones, the greatest change in transition rates was observed during the initial days of culture. They showed the strongest mesenchymal-mesenchymal (M-M) state transition rate (0.5786 ± 0.07) followed by that for the epithelial-epithelial (E-E, 0.5526 ± 0.09), epithelial-mesenchymal (E-M, 0.5096 ± 0.088 and mesenchymal-epithelial transition (M-E, 0.505 ± 0.12). Similarly, for the UMUC3 cell line, in the cells derived from E clones, epithelial-mesenchymal (E-M, 0.6364 ± 0.70) rate was highest as compared to all other transitions (E-E, 0.3838 ± 0.49; M-E, 0.4528 ± 0.33; M-M, 0.6314 ± 0.48). Nonetheless, over 2–3 generations, none of the subpopulations maintained its original seeding state; rather all states were reconstituted showcasing bidirectional transition between epithelial and mesenchymal-like cell states in vitro and suggesting a tendency towards maintaining the original equilibrium between the states in the long term.

### Transcriptome-level differences between cell states suggest EMT

To examine transcriptomic makeups associated with the observed cell states and identify gene regulatory network underlying the state transition dynamics, we performed single cell RNA sequencing of populations of cells from the T24 cell line derived from E and M-like clones. UMAP analysis revealed that the transcriptomes of the T24 cells of the same (E or M) phenotypic states tightly clustered, while minor populations of cells therein clustered with the cells of different phenotypic states (Fig. 2f, Supplementary Fig. 2f, g) – indicating that phenotypically similar cells also have similar transcriptomes. Single sample GSEA scores for bladder cancer subtype-related genesets suggested that majority of the cells from both the E and M populations in the T24 cell line were basal type, indicating that the cellular clusters are not due to cancer subtypes (Fig. 2g). Next, we analyzed cancer hallmarks and pathway signatures of the cellular clusters. Epithelial and mesenchymal marker genes *CD24* and *CDH2* had anti-correlated expression; *CD24* upregulated in E and *CDH2* upregulated in M cells (Fig. 2h). Furthermore, M cells had relatively higher EMT and stemness gene signatures than E cells– which is consistent with their respective phenotypic characteristics and *CD24*/*CDH2*-based observations (Fig. 2g). The T24 cell populations could be resolved into further subclusters (cluster 0-4) that may reflect potentially intermediate transcriptional states (Supplementary Fig. 2g). The cluster 0 comprised of majorly M cells and presented with the highest EMT and stemness gene signatures (Supplementary Fig. 2h); but the clusters 1 and 3 had major contribution from E cells, representing 2 cell sub-populations with different transcriptional states. While the cluster 3 cells showed reduced EMT and stemness phenotypes, the cluster 1 cells showed features of M cells with comparatively high EMT and stemness traits, albeit less than that of the cluster 0, suggesting E cells also harbors some transient cells which are poised for phenotypic modulation to M transcriptomic state by losing their epithelial identity, while acquiring mesenchymal phenotypic markers. Interestingly cluster 4, with comparable contribution from both M and E cells showed the lowest expression of EMT and stemness features suggesting they might be highly differentiated terminal cells. We also used differential expression testing and gene set analyses and found that mediators of EMT like TGFB and TNFA pathways and EMT pathway are upregulated in in cluster 0 but TNFA signaling downregulated in cluster 4^37–39^ (Supplementary Fig. 2i), consistent with our finding that M cells show highest EMT phenotype. These results indicate that the transcriptional and phenotypic clustering identify interpretable cellular sub-populations with some variation in EMT characteristics. In contrast, the overall transcriptional makeup of UMUC3 cell line was different. UMUC3 single cell RNA sequencing data identified substantial populations of cells with basal and luminal subtype signatures. Based on the EMT signatures we observed subpopulations of cells with E or M-like transcriptomic states; cluster 0 showed both high EMT and stemness signature, on the other hand clusters 3 and 4 had low scores for EMT and stemness signatures (Fig. 2i, Supplementary Fig. 2j, k). Again, the transcriptional states were not directly attributable to the subtype annotation, consistent with that observed in the T24 cell line, indicating that emergence of the E and M-like transcriptional states are not due to subtypes. Taken together, our imaging and single-cell RNA analyses of two bladder cancer cell lines with distinct characteristics reveal that, (i) cellular transcriptional and phenotypic annotations overlap, indicating transcriptomic underpinning of morphological variations, (ii) cells are segregated in predominantly E and M-like states, with minor intermediate cell populations and (ii) during in vitro evolution, heritability and transition between E and M-like cell states contribute towards population-level cell state plasticity, reconstitution potential, and overall growth dynamics.

### Regulatory network underlying transcriptional state dynamics

**Fig. 3:**
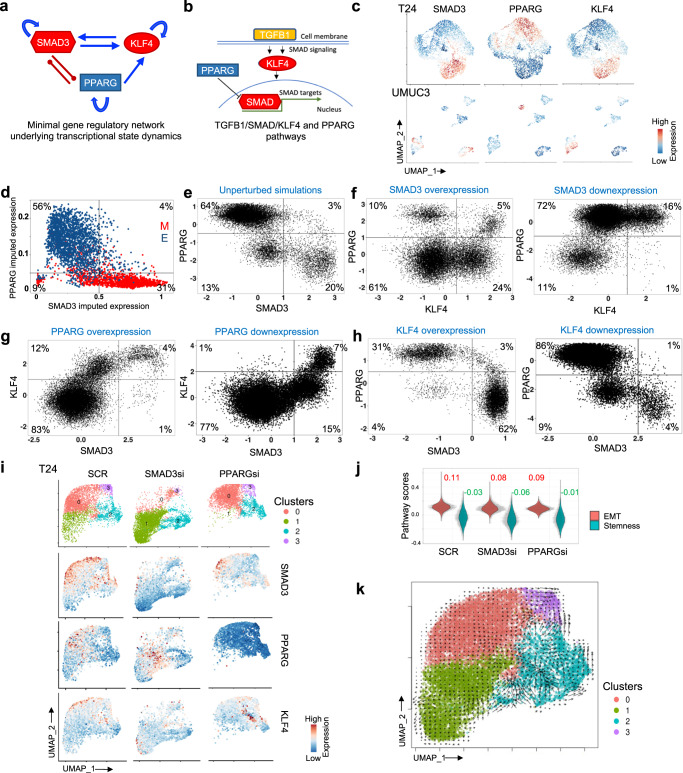
Transcriptional regulatory network associated with cell states. **a** Data driven prioritization of a minimal gene regulatory network underlying the transcriptional state dynamics. Blue lines and arrowheads represent the gene activation; red lines and blunt heads represent gene inhibition. **b** A schematic representation of TGFB1/SMAD/KLF4 and PPARG pathways are shown. **c** UMAP plots of single cells from M and E transcriptomic states from T24 and UMUC3 cell lines, colored by expression of regulatory marker genes. **d** Scatter plot showing expression of indicated genes in M and E cells. Imputation was used to avoid zero-inflated representation. **e**–**h** Scatter plot showing scores for all RACIPE solutions. **i** UMAP plots showing changes in transcriptional states of T24 cells upon targeted perturbation of the genes in the regulatory network, wherein SCR indicates control cells and SMAD3si and PPARGsi indicate SMAD3 and PPARG siRNA treated cells, respectively. The panels below show patterns of activities of the genes using a color gradient. **j** Violin plots showing expression of indicated oncogenic signatures following siRNA treatment with median values indicated on top of the plots. **k** Single cell RNA velocity estimates for individual cells in targeted perturbation experiment with arrows indicating the extrapolated direction of transition to transcriptional states projected onto the UMAP plot. Cells are colored by their cluster IDs. Lengths of the arrows indicate the velocity of transition between the clusters.

We next sought to identify the gene regulatory network that governs transition between the transcriptional states observed above. We constructed a closed, minimalistic, core gene regulatory network based on a data-driven approach by (i) selecting known transcription factors (TFs) that are master regulators, and associated with epithelial and mesenchymal characteristics, (ii) inferring their activities based on the changes in expression levels of their targeted genes in the E and M-like transcriptional clusters, (iii) identifying activating and/or repressing regulatory interactions among them and (iv) iteratively identifying the minimal set of TFs that satisfy these criteria. We identified a core network of 3 key TFs - SMAD3, KLF4 and PPARG, that satisfy the key properties, and also play more central roles in EMT related regulation within a broader network of transcriptional regulators (Fig. 3a, Supplementary Fig. 3a)^40,41^. PPARG agonists could activate the expression of *KLF4* by binding to the PPAR binding site in the *KLF4* promoter^42^. It is also known that TGFB1 signaling can suppress the expression of *PPARG* via SMAD binding^43^. On the other hand, PPARG can suppress the activity of TGFB1/SMAD3 signaling^44^ thus forming a toggle switch topology with SMAD3. KLF4 has been known to activate the mesenchymal program in cancer stem cells through the increased activity of the TGFB1/SMAD signaling^45^. Similarly, SMAD3 and upstream TGFB1 signaling can activate the expression of *KLF4* via the HIF1a pathway^46,47^, thus establishing a positive feedback loop between *KLF4* and *SMAD3*. All three of these transcription factors are known to self-activate their own expression via auto-regulatory loops either through direct or indirect means (Fig. 3b). Levels of *SMAD3* and *KLF4* were higher in the M cells while the level of *PPARG* was particularly high in the E cells of the single cell data (Fig. 3c)^44,48^. We also observed inverse relationship between expression of *SMAD3*/*KLF4* and *PPARG* in both T24 and UMUC3 cells (Fig. 3c, Supplementary Fig. 3b). The regulatory network is analogous to the MET/SMAD3/SNAIL/miR-323a-3p circuit reported in the context of epithelial–mesenchymal transition in bladder cancer^49^. PPARG is involved in selective suppression of *SNAIL*, while KLF4 and miR-323a-3p are involved along the STAT3 axis^50,51^, which has extensive crosstalk with SMAD3^52^. While miR-323a-3p cannot be directly detected in single cell RNA sequencing data, and the prior work on MET/SMAD3/SNAIL/miR-323a-3p network lacked data on dynamic state transition at single cell resolution, consistency of the observations supported our network inference. Furthermore, it underscores the importance of the regulome of the transcriptional states beyond that of the individual genes in isolation, a key focus of this study. Next, we simulated the gene regulatory network in Fig. 3a using the computational framework RACIPE to evaluate the steady state solutions allowed by the network topology^53^. In the simulation we observed that– *PPARG* high *SMAD3* low phenotype (64%) and *PPARG* low *SMAD3* high (20%) were the most predominant states, representing the equivalent of E and M-like transcriptional states that are seen experimentally; moreover, the values are qualitatively like that inferred from the single cell data (Fig. 3d, e, Supplementary Fig. 3c). The other two transcriptional states that are present in simulation in low abundances are *PPARG* low *SMAD3* low (13%) and *PPARG* high *SMAD3* high states (3%); the double positive and the double negative phenotypes were even less abundant in the single cell RNA sequencing data.

### Transcriptional state dynamics under regulatory network perturbations

We performed in-silico and experimental targeted perturbation experiments to establish whether silencing of key regulatory genes can help alter the transcriptional state equilibrium in mixed population of cells, by pushing the cells towards one or the other transcriptional states. First, in silico overexpression of *SMAD3* using RACIPE framework led to an increase in prevalence of M state from to 20% to 84% and also variable expression of *KLF4* across the entire dynamic range - suggestive of permissive occupancy of transcriptional states related to naïve and primed pluripotency and possible intermediate states in-between (Fig. 3f, Supplementary Fig. 3d); contrary findings were seen upon *SMAD3* under-expression. Upon *PPARG* overexpression, the prevalence of the E state increased from 64% to 91% along with concurrent downregulation of *SMAD3* and *KLF4*. Opposite trends were observed when *PPARG* was under-expressed, and the relative abundance of the cells in E state was reduced to 8% of the total steady state population (Fig. 3g, Supplementary Fig. 3e). Lastly, when *KLF4* was overexpressed, M state characterized by high expression of *SMAD3*, became more prevalent (20% to 62%) while its downregulation reduced that to 4% (Fig. 3h, Supplementary Fig. 3f). These simulations suggest that regulation of *SMAD3*, *PPARG* and *KLF4* can control the prevalence of E and M-like transcriptional states and modulate their transition dynamics. Next, treatment with gene-specific siRNAs targeting *SMAD3* and *PPARG* (SMAD3si, PPARGsi) resulted in systematic downregulation of *SMAD3* and *PPARG* respectively in T24 cells, as observed in scRNAseq data (Fig. 3i, Supplementary Fig. 3g; see Method for details). *KLF4* had very low baseline expression and is induced by both *SMAD3* and *PPARG*; hence, no *KLF4* siRNAs were used. The downregulation of *SMAD3* indeed increased prevalence of E state over M state, and led to reduced EMT and stemness, (Fig. 3i, j, Supplementary Fig. 3h, i). Expression of EMT marker genes *CD24* and *CDH2* also showed consistent pattern (Supplementary Fig. 3j). RNA velocity analysis indicated that cells in the cluster 2, which had representation from all treatment types and showed the lowest EMT score, were likely in the transient transcriptional states, and cells from other clusters were poised to head towards this state (Fig. 3k, Supplementary Fig. 3g). Most of the other cells maintained steady transcriptional states. Altogether, our experimental observations are consistent with that from the RACIPE modeling analysis and suggest that SMAD3 and PPARG form a minimally sufficient regulatory network with KLF4, that can explain the transcriptional state dynamics. While we do not rule out that other regulatory factors may impact transcriptional state dynamics, targeted perturbation of this minimal network altered the transcriptional state equilibrium in a predictable manner.

### State dynamics under nutrient starvation in vivo and in vitro

**Fig. 4:**
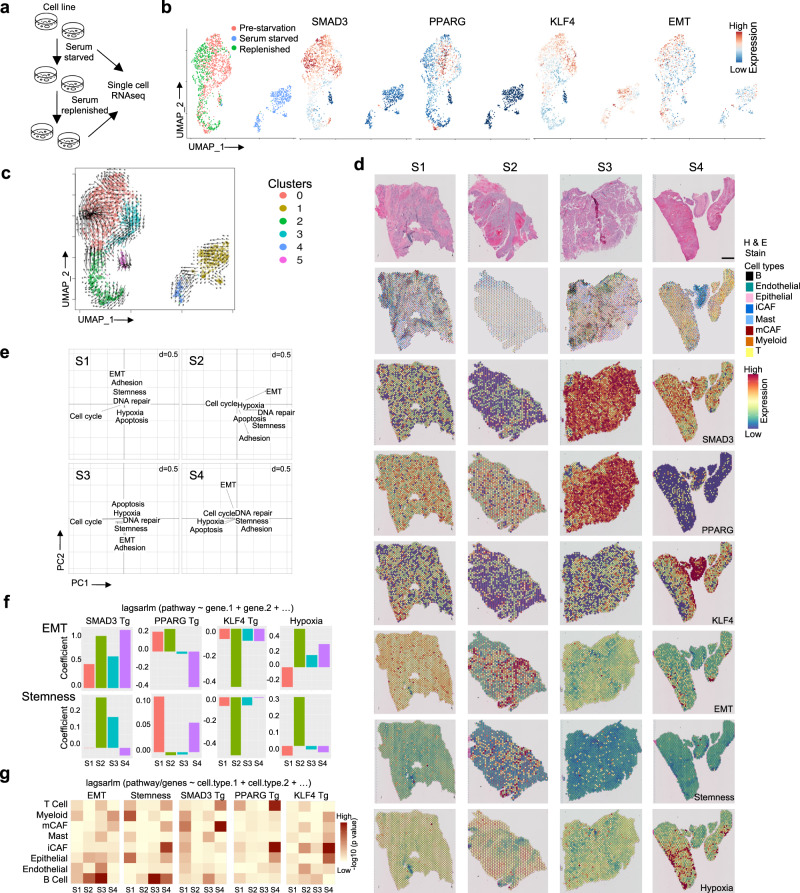
Transcriptional states in tumor microenvironments. **a** A schematic representation showing serum starvation and replenishment of T24 cell line model system. **b** UMAP plot showing transcriptomic changes in the cell populations before and after serum starvation, and after replenishment; the panels show patterns of activities of genes or pathway signature using a color gradient. **c** Single cell RNA velocity estimates for individual cells in nutrient starvation and replenishment experiment with arrows indicating the extrapolated direction of transition to transcriptional states projected onto the UMAP plot. Cells are colored by their cluster IDs. Lengths of the arrows indicate the velocity of transition between the clusters. **d** Hematoxylin and eosin (H&E) staining, spatial scatter pie plot displaying inferred cell type composition on each spatial location from deconvolution and expression scores of genes and relevant oncogenic pathways from spatial transcriptomics data for the four bladder cancer specimens, indicated as S1-S4. Scale bars represent 1 mm. **e** Spatial principal component analysis (sPCA) plot shows the loading of different cell types along the first two principal axes. **f**, **g** Pathway or gene activity modeled as a function of the estimated abundance of the cell types or function of expression of target genes (Tg) of relevant genes and pathway using multivariate regression with a spatial lag to account for spatial autocorrelation, with barplots showing coefficients and heatmap showing p-value associated with coefficients for the cell types from spatial transcriptomics data for the four bladder cancer specimens, indicated as S1-S4.

At the time of surgical resection, UBC patients are often presented with large tumors with a nutrient starved, hypoxic core^54,55^, where stress conditions are known to modulate tumor cell phenotypes – that motivated us to use the T24 cell line model to examine the effects of serum starvation on transcriptional state dynamics. We seeded each well of 24-well plates individually with M cells, and serum deprived the cells overnight and then replenished them with fresh serum containing medium to study state transition and reversal of phenotypes (Fig. 4a). We profiled gene expression data at single-cell level and observed that serum starved cells overall steer towards a low M and low *SMAD3* state (Fig. 4b, Supplementary Fig. 4a). From 6 major clusters identified, cluster 3, which was majorly comprised of pre-starvation cells but with some fraction of serum replenished (0.027) and serum starved cells (0.004), presented with the highest EMT and stemness scores, further corroborating some cells do inherently confer a survival advantage compared with other cells, and others showing rapid extinction (Fig. 4b, Supplementary Fig. 4b, c). Cluster 0 showed high expression of EMT, low apoptosis and hypoxia, with high *SMAD3* levels as assessed through expression levels of a panel of gene signatures (Supplementary Fig. 4c–e). A minor population of cells in the pre-starvation and replenished culture also clustered with serum starved cells – suggesting that this phenotype may be present, albeit sparingly, in nutrient-rich conditions. We further analyzed RNA velocity of single cells and observed the highest transitions in serum supplemented cluster 0 and lowest in serum starved cluster 1 (Fig. 4c). Overall, these data support the notion that nutrient limitation affects the cell state transition dynamics and may impact the overall cell population-level phenotype. Moreover, a minor cell population with M state may present a survival advantage during serum starvation and could reconstitute growth and phenotypic diversity upon nutrient replenishment. Furthermore, tumor microenvironment influences regulation of gene expression, nutrient availability, and tumor cell phenotypes including EMT. Cancer-associated fibroblasts (CAFs) promote the mesenchymal phenotypes of tumor cells^10,56^. Hence next we analyzed 10X spatial transcriptomic data from 4 UBC samples at different stages of aggressiveness to evaluate physiological relevance of our in vitro observations. Samples included Sample 1 (S1); a high-grade invasive urothelial transitional cell carcinoma with lymph node metastasis but no distant metastasis, Sample 2 (S2); a high-grade invasive localized urothelial transitional cell carcinoma without lymph node or distant metastasis, Sample 3 (S3); a high-grade non-invasive papillary urothelial carcinoma without lymph node or distant metastasis, Sample 4 (S4); a high-grade invasive urothelial transitional cell carcinoma with squamous differentiation, and negative for lymph node or distant metastasis. We annotated the four samples based on H&E staining and cell type compositions and colored the spatially annotated barcodes in the tissue samples accordingly, and then estimated expression of *SMAD3*, *KLF4* and *PPARG*, and pathway-level scores for EMT, stemness and hypoxia (Fig. 4d, Supplementary Fig. 4f). *SMAD3* and *PPARG* expression showed complementary patterns, while *SMAD3* and *KLF4* showed spatially similar patterns in invasive tumors S1, S2 and S4. In non-invasive tumor S3, all three regulatory genes were highly expressed. We found substantial inter-tumor differences and also intra-tumor spatial variations in EMT signature. EMT and stemness signature were consistently a major principal component of spatial biological variations regional variations within tumor tissues as observed in the spatial principal component analysis (sPCA) plots (Fig. 4e). Nonetheless, EMT and stemness signatures were spatially associated, especially in advanced tumors. Next, we applied a published network graph-based multivariate analysis^57^ to determine whether spatial variation in EMT signature can be modeled based on nutrient limitation and expression of the genes in the identified regulatory network after accounting for spatial patterns of autocorrelation. In brief, since spatial transcriptomics had zero-inflated RNAseq data and activities of TFs can be inferred from expression of their target genes, we used a multivariate regression model including expression signatures of the *SMAD3*, *KLF4* and *PPARG* target genes with a spatial lag to account for spatial autocorrelation along the neighborhood graph (Fig. 4f). We acknowledge small sample size, limited regional variation in hypoxia within profiled regions, and sparse spatial transcriptomic data, and therefore conservatively interpret the results. Though there were substantial inter-tumor variations in the patterns of spatial heterogeneity in tumor microenvironment characteristics, the proportion of variance in pathway-level scores estimated by spatial autoregressive parameters, explaining the abundance of different cell types was modest. Nonetheless, spatial multivariate analysis indicated that hypoxia and *SMAD3* target genes expression were positively associated with EMT, which, despite weak effect sizes, were in line with our observations in vitro. Next, we modeled regional variation in the pathway activities based on regional abundances of cell types. In tumors S2 and S3 the EMT signature was significantly associated with epithelial cell abundance, while stemness signature in the other two samples (Fig. 4g, Supplementary Fig. 4g).

### Radiation treatment-associated transcriptional state dynamics in vitro

**Fig. 5:**
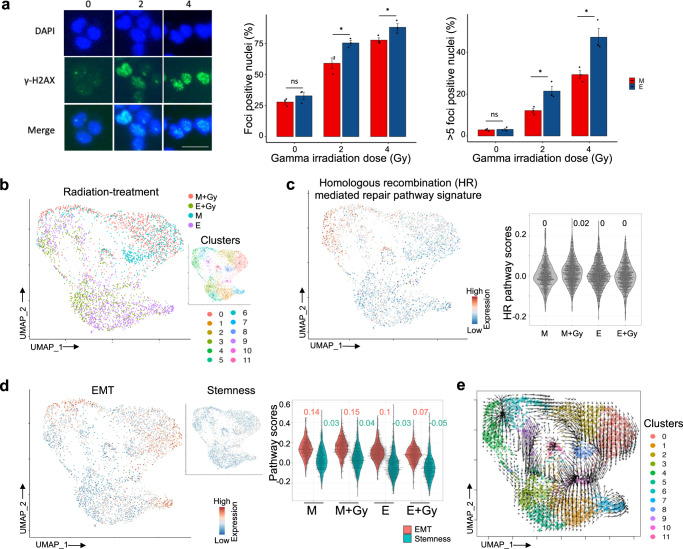
Impact of radiation on transcriptional profiles. **a** Representative images and quantification of γ-h2ax foci upon irradiation in T24 cells. DAPI was used as a nuclear counterstain. Scale bars indicate 20 μm. Barplots show differences in foci positive nuclei upon irradiation between cells at E and M states. Error bars represent standard deviation. P-value was calculated using t-test wherein *p value < 0.05. **b** UMAP plot of single cell RNAseq data showing single cells from gamma-irradiated and non-irradiated cells initiated from E and M states. The inset showing the same plot, annotated by cluster IDs. **c** UMAP and corresponding violin plots show activities of homologous recombination mediated DNA repair pathway in the groups of cells. **d** UMAP and corresponding violin plots show the activities of EMT and stemness gene signatures in the groups of cells, with median values on top of plot. **e** Single-cell RNA velocity estimates for individual cells in gamma-irradiated and non-irradiated M and E cells with arrows indicating the extrapolated direction of transition to transcriptional states projected onto the UMAP plot. Cells are colored by their cluster IDs as in (b). Lengths of the arrows indicate the velocity of transition between the clusters.

Since a treatment modality for patients with invasive UBC is radiation, typically concurrent with chemotherapy^2^, we examined whether response to radiation treatment differed between the transcriptional states, and whether transcriptional state dynamics could confer transient resistance at the level of tumor cell populations. We first compared the effect of gamma irradiation (Gy) on DNA damage and cell survival in T24 cell line in vitro. DNA double-strand breaks, measured by the γ-H2AX assay, were repaired more rapidly in the cells with M than in E phenotypes at 24 h after the treatment (Fig. 5a, Supplementary Fig. 5a). We observed a significantly smaller number of foci in M cells as compared to E cells with irradiation with 2 and 4 Gy. A relative increase in foci number was exhibited at 4 Gy for both M and E phenotypes. This shows cells with M and E states have different DNA damage response and repair characteristics, with M exhibiting a more rapid and efficient repair than the E state cells. Therefore, the risk of accumulation of replication errors and mutations induced by DNA damaging agents appears lower in M type cells, which is consistent with published reports^58,59^. However, these cells were capable of transforming into E-like states when propagated and strived towards maintaining a cell population-scale equilibrium in transcriptional states. This supports a model where different transcriptional states confer different radio-sensitivity. However, owing to adaptive transcriptional state dynamics, cell population-level radio-resistance emerges instead of one population outcompeting the other. Next, we investigated transcriptomic dynamics in radiation treated T24 cells at single cell resolution to understand the underlying molecular changes. We performed single-cell RNA sequencing of colonies derived from M and E cells at 24 h postirradiation with 2 Gy and compared that with untreated cells (Fig. 5b, Supplementary Fig. 5b). Overall DNA damage response pathway (DDR) was upregulated in E cells, but homologous recombination pathway (HR) was higher in M cells and lower in E cells post-irradiation, increasing the DNA damage tolerance and prolonging the survival of M type cells (Fig. 5b, c, Supplementary Fig. 5c, d). We observed multiple subpopulations with distinct phenotypes; cluster 11 showed the highest HR scores, to which cells in M state contributed the most, with and without Gy treatment, apart from cluster 4 and 6 which were enriched in cell cycle genes. Transcriptional states associated with M were also associated with up-regulated EMT and stemness signatures (Fig. 5d). Furthermore, clusters 2 and 8 with major contributions from treated and untreated control cells in M state respectively, presenting with EMT and stemness phenotype and high *SMAD3* expression (Supplementary Fig. 5e). However, cluster 7 harboring mesenchymal phenotype and low DDR, were majorly contributed by untreated E cells (0.76) and some irradiated E cells (0.17). Cluster 10 showed a similar pattern to cluster 7. Our results suggest cells in M state with high mesenchymal phenotype most likely tolerate and survive radiation to repopulate the tumor later. The clonal dynamics in E populations were more complex. Jointly analyzing the datasets from cells in M and E states, we observe that overall extreme cell states, which include cells with very low EMT score in the former and very high EMT score in the latter, went extinct upon radiation treatment, while the cells with moderate EMT scores persisted. The results indicate that the *SMAD3* and *KLF4* high M state could reduce DNA damage and promote cell survival and EMT. Moreover, *SMAD3* has been shown to increase radiosensitivity upon downregulation^60,61^. *KLF4* has also been shown to be upregulated after chemotherapy, potentially mediating therapy-related stemness phenotype^62^. On the other hand, *PPARG* expression promotes radiosensitivity^63^. Thus, our observations are consistent with the published biological functions of the gatekeepers of the transcriptional state dynamics. Analyzing velocity field in UMAP embedding of gene expression, we further observed that E cells were poised to transform towards cluster 3 - a low EMT state (Fig. 5e). However, despite having limited connectivity between M and E clusters, cells from both phenotypes were transcriptionally transforming towards cluster 10, which is an intermediate EMT state and stemness phenotype, and low DNA damage response activity (DDR). Taken together, we argue that although *SMAD3* and *KLF4* promote radioresistance and *PPARG* radiosensitivity, radiation treatment likely eliminates extreme transcriptional states, and/or rather surviving cells converge towards transient equilibrium dominated by intermediate EMT scores, as a radiation response.

### Clinical relevance of the regulatory network

**Fig. 6:**
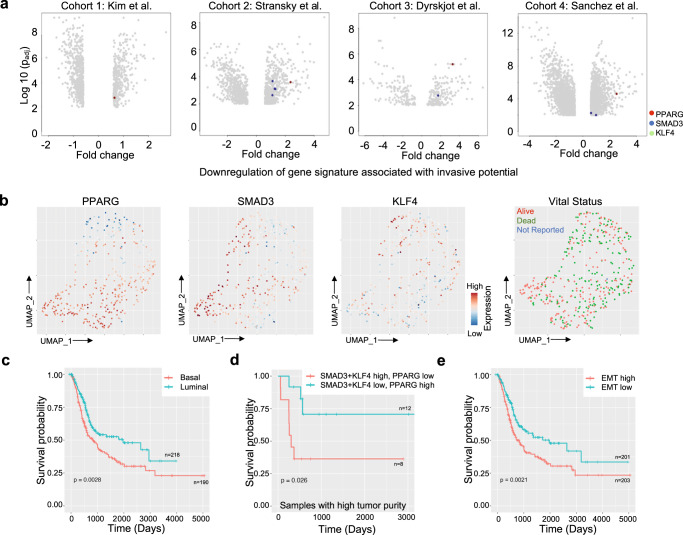
Prognosis in urothelial bladder cancer patients. **a** Volcano plots showing differential expression of genes, including those in the regulatory network in bladder tumors with different invasive potentials in four independent bladder datasets. Positive and negative values of fold change indicate down- and upregulation of invasive potential signatures, respectively. Each grey dot is a differentially expressed gene. SMAD3 and PPARG were annotated, but KLF4 was not among the significantly differentially expressed genes. **b** UMAP plots of the samples in the TCGA-BLCA cohort, colored by expression levels of SMAD3, PPARG, and KLF4 and the vital status. **c**–**e** Kaplan-Meier survival analysis of the bladder cancer samples from TCGA-BLCA cohort according to (**c**) subtype annotation, (**d**) differential expression of the genes in the regulatory network in the samples with high tumor purity, and (**e**) predominant transcriptional states. *p* values were calculated using Log-rank test.

We examined the effects of the gene-trio involved in the regulatory network underlying the transcriptional state dynamics on prognosis and overall survival in the UBC patients. Analyzing microarray-based gene expression data for bladder cancer study^64^, we found *PPARG* is strongly associated with a non-muscle invasive phenotype as compared to muscle-invasive phenotype in multiple cohorts, while *SMAD3* has borderline contrarian pattern in those (Fig. 6a), which might be due to the fact that core EMT regulators are often co-expressed in various combinations in order to orchestrate complex EMT programs depending on the specific biological context. Furthermore, in the TCGA-BLCA cohort, *PPARG* expression was a differentiating feature between patients’ samples in UMAP projections and to a lesser extent *SMAD3* and *KLF4* expression (Fig. 6b). We further performed Kaplan–Meier analysis to evaluate the prognostic value of prevalence of the transcriptional states and the associated gene regulatory network (Supplementary Fig. 6a). Tumors with above median *PPARG* expression had significantly longer overall survival (Supplementary Fig. 6a, *p* = 0.0059, median survival of *PPARG*-high group: 1163 days (~3.1 years), median survival of *PPARG*-low group: 859 days (~2.4 years)). *KLF4* upregulation was associated with poor survival, but this effect was not significant and *SMAD3* showed some ambiguity. All three genes together- *SMAD3* and *KLF4* high, and *PPARG* low expression, were associated with poor survival, although not significant (Supplementary Fig. 6a). We then used deconvolution algorithms to estimate cell-type proportions from the bulk tumor gene expression data in the TCGA-BLCA cohort as cellular compositions of tumors might be different. Supplementary Fig. 6b, c shows dominant cell types in each sample inferred from deconvolution and association among different cell types; the extent of immune infiltration showed inverse correlation with the epithelial phenotype. The tumor microenvironment harbors both immune-suppressive and activating cells, and the tumor infiltrates are highly heterogeneous depending on the specific cancer type. Immune-rich phenotypes also presented with decreased and significant hazard ratio (*R* = 0.67, *p* = 0.028), suggesting better prognosis (Supplementary Fig. 6d). We then correlated the cell fraction data with clinical information from TCGA-BLCA cohort and noticed that the cell type abundance was altered greatly across the molecular subtypes. Upon subtype classification and cell type estimations, luminal tumors had the best prognosis; and basal tumors with high immune infiltration had worse prognosis (Fig. 6c). It has already been demonstrated that PPARG pathway activation is associated with the luminal intrinsic bladder subtype^65^. Also, tumors with high EMT scores are mostly associated with high immune infiltration; however, high EMT and reduced infiltration of immune cells and vice versa is also observed, albeit in few tumors (Figs 1, 6). Interestingly, in tumors high tumor abundance, *SMAD3* and *KLF4* high, and *PPARG* low expression, were significantly associated with poor prognosis, albeit small sample size (Fig. 6d). When the survival analysis using EMT signature was performed, there was a significant survival difference in tumor patients (Fig. 6e, *p* = 0.0021, median survival of EMT-high group: 823 days (~2.2 years), median survival of EMT-low group: 1348 days (~3.7 years)). Altogether, our results suggest that EMT signature has a prognostic significance in UBC patients and supports a model that tumors dominated by transcriptional cell states with low EMT and high *PPARG* expression have better survival.

## Discussion

We analyzed single-cell and spatial transcriptomics data from urothelial bladder cancer (UBC) specimens and used live cell imaging and single-cell genomics in bladder cancer cell lines to characterize transcriptional heterogeneity in bladder cancer, establish its mechanistic basis, and understand its clinical importance. Our results show that intra-tumor transcriptomic heterogeneity in UBC is, at least partly, due to co-existence of tumor cells having epithelial- and mesenchymal-like transcriptional states. Moreover, recurrent and reversible transition between the transcriptional states contribute towards dynamic, nongenetic heterogeneity both in vitro and in vivo^13,66,67^. Our integrative analyses identify several key characteristics of dynamic transcriptional heterogeneity in urothelial bladder cancer.

First, detection of these transcriptional states recurrently in different patients and different subclones within any patient indicates that, the phenotypes are partially independent of genetic variations, subtype annotation, and that transition between these recurrent transcriptional states might be frequent within and across the branches of tumor phylogeny – an emerging pattern across several cancer types^17,21,68,69^. While cancer is considered a genetic disease, these findings collectively point towards the nongenetic basis of the dynamic emergence of an important cancer hallmark.

Second, transcriptional state dynamics involving epithelial-mesenchymal transition appear to be regulated by a core regulatory network. Our data-driven approach identifies SMAD3, KLF4 and PPARG as regulatory markers of the spontaneous transcriptional state dynamics^70–72^. KLF4 is a Yamanaka factor important for pluripotency, while SMAD3 is essential for TGFB1 signaling, and PPARG has complex roles in tumor metabolism and immunity; they have known roles as positive and negative regulators of EMT and bladder carcinogenesis^40,41,44,45^. We note that the complex regulation of transcriptional states involves chromatin remodeling, as well as complex changes in metabolism, signaling, and other cellular processes; thus, it is not surprising that several other factors^49,73–78^ could also influence epithelial-mesenchymal transition dynamics. However, at the pathway-level, these results appear to converge implicating TGFB and MET pathways, and metabolism in regulating EMT-associated transcriptional state dynamics^79–82^. Our results are consistent with prior reports focusing of the role of MET/SNAIL/SMAD3/miR323a-3p circuit^49^ on EMT dynamics, underlining the importance of the complex regulon governing the transcriptional state dynamics, beyond the classic oncogenic functions of these genes individually. Our data-driven unbiased analyses in the model system, with consistent observations in the genetic and extrinsic perturbation experiments, as well as in the single cell and spatial transcriptomics data from human UBC tumors, and survival data analyses suggest that the gene regulatory network involving SMAD3-KLF4-PPARG could be minimally sufficient to modulate the transcriptional state dynamics in vivo and in vitro, serving as clinically informative biomarkers.

Third, despite heritability of transcriptional states, tumor cell populations show inherent propensity to attain and maintain dynamic equilibrium among the transcriptional states^83,84^. This phenotypic plasticity likely offers evolutionary advantages and adaptability to tumor cell populations, especially under stress. Transition of cancer cells across a continuum of states in response to therapy is emerging as a potential mechanism to develop drug tolerance^23,85–87^ and our data suggests that exposure of cells to stress could induce transition of some mesenchymal-low subpopulations into mesenchymal-high subpopulations, with SMAD3-KLF4-PPARG inducing the phenotypic switching. Our simulation and experimental results show that feedback loops within this core network can give rise to co-existence and stability of different transcriptional states. Microenvironmental cues such as nutrient starvation and clinical management strategies such as radiation affect cell state transition dynamics, selecting for a transiently resistant phenotype and then dynamic population-level reconstitution of growth and heterogeneity upon removal of stress condition. Bladder tumors are often large and nutrient-starved; our spatial transcriptomics analyses indicates that transcriptional states of tumor cells show regional autocorrelation and may be associated with microenvironmental contexts. Our spatial analysis also revealed that different transcriptional states with differential EMT potency are localized in different niches and contribute to the spatial organization of tumor subpopulations in patients with UBC^88,89^. Analysis of tumor immune microenvironment of bladder cancer at the single-cell level by Chen et al.^10^ revealed distinct subpopulations and functional heterogeneity of cancer-associated fibroblasts (CAFs). Our single-cell and spatial transcriptomics analyses support the idea that transcriptional state heterogeneity and plasticity of tumor cells in the microenvironment are dominated by E and M states and could be modulated by local microenvironmental contexts. It is possible that tissue-level coordination of transcriptional state dynamics may involve inter-cellular signaling and non-autonomous mechanisms; for instance, it was shown that urothelial cells undergo epithelial-to-mesenchymal transition after exposure to muscle-invasive bladder cancer exosomes^75^.

Lastly, intra-tumor heterogeneity and transient resistor phenotypes are difficult to target^90,91^. In the context of radiation treatment, initial perturbation of transcriptional-state dynamics and selection of a transiently resistant phenotype, followed by reconstitution of heterogeneity present potentially complex challenges for clinical management of UBC^92–94^. Somatic evolution in tumor is classically described as Darwinian process, but these findings contribute to the debate about Lamarckian adaptation during treatment^95–97^. Further studies are necessary to establish detailed molecular mechanisms. Tumor subtype classification and targeting recurrently mutated cancer genes has been a mainstay of classic cancer research and therapeutics^98,99^, but limited efficacy due to emergence of resistance remains a problem^4,100,101^. Intra-tumor heterogeneity and plasticity present substrates for evolvability and treatment resistance in urothelial bladder cancer, and other cancer types^102,103^. So far, limited clinical management strategies are available to target or even contain intra-tumor heterogeneity^22,104,105^. Our observations indicate that, key biomarkers of the regulome of the transcriptional state dynamic heterogeneity in cancer could be identified and targeted to alter the overall cell population-level phenotype and potential for treatment resistance, which may modulate intra-tumor heterogeneity itself and help formulate mono- or combination therapy for effective management of heterogeneous tumors.

## Methods

### Bladder cancer cell lines

Bladder cancer cell lines T24 and UMUC3 were purchased from the American Type Culture Collection (ATCC, USA) and were regularly tested for mycoplasma contamination. They have different growth potentials and were cultured in Dulbecco’s modified eagle’s medium (D6429, Sigma-Aldrich) supplemented with 10% fetal bovine serum (97068085, VWR) and 1% Penicillin-Streptomycin solution (97063708, VWR), at 37°C in a humidified incubator with 5% CO_2,_ and harvested using trypsin-EDTA solution (25200056, GIBCO) or rubber-tipped cell scraper.

### Monoclonal selection and secondary cloning

Two methods were used to characterize the clonal composition of bladder cancer cell lines- cloning rings and limited dilution. For plating by limited dilution, cells were plated at very low cell densities (<1 well per well in 96 well plates) with 50% conditioned medium and after a day when the cells were attached, wells containing a single cell were marked; empty wells and wells containing >1 cell were excluded. Seven days following plating, colonies derived from single cells were designated as holo-, mero-, and paraclones by morphological characterization. The colonies were grown to confluence and transferred to twenty-four-well plates where they were maintained until near ~80% confluent, at which time they were subjected to various treatments. For cloning ring method, ~20 cells per seeded per 100 mm dishes and monitored for clonal expansion. Once colonies reached density of at least 50 cells, colonies were isolated from the culture dish using clonal discs. Briefly, colonies from the parental population were classified as holo-, mero-, and paraclones based on colony morphology and well-spaced colonies of each type were selected, ring-cloned and further propagated until reaching ~80% confluency. The number and types of each type of colony produced were determined for clonogenicity assessment. Automated cell counter Vi-CELL cell analyzer (Beckman Coulter) was used to determine the cell viability of each colony type.

### Incucyte time-lapse microscopy and analysis of clonal growth

Cells were plated at low cell densities, in the range of 25-100 cells per well in 24-well plates and allowed to give rise to colonies over a period up to 2 weeks. Colony growth from single cells/few cells was monitored using the Incucyte® Live Cell Analysis System to capture time-lapse phase contrast images of each well. Incucyte images were taken at intervals of 2 hours and each colony was tracked from when cell first adhered to the plate to the end of the growth period. The number of cells from which the colony developed, number of colonies derived, the merging of colonies and the colony morphology were estimated. After cells reached ~70-80% confluence, the clones were given indicated treatments and returned to Incucyte incubator for image tracking. The Incucyte system was also used to measure progressive wound closure in scratched wells over time for migration assay and generate confluence graphs for proliferation assay.

### Knockdown of gene expression

ON-TARGETplus siRNAs were used for knockdown of *SMAD3* (SMAD3si) (J-020067, Horizon Discovery), *PPARG* (PPARGsi) (J-003436, Horizon Discovery) and Scrambled (SCR) was used as a negative control (D-001810, Horizon Discovery). For gene expression knockdown, cells were transfected with the indicated siRNAs at a final concentration of 50 nM using Lipofectamine (13778075, Thermo Fisher Scientific) following standard protocols for cell lines.

### Serum starvation and irradiation treatment

After cells reached ~80% confluence, the medium was replaced, and the cells were irradiated with 2 and/or 4 grays (Gy) of γ-radiation using Gammacell 40 Extractor (MDS Nordion) at an average dose rate of 91 cGy/min. Following irradiation, cells were immediately returned to the incubator and allowed to recover for 24 h. For serum starvation, the growth medium was replaced with medium completely devoid of FBS and cells were cultured overnight (~15 h), after which serum was re-supplemented, and cells were allowed to recover for 24 h. Parental cells and untreated monoclonal cells were used as controls and were counted and passaged under the same conditions without irradiation or serum starvation. Recovered cells were then harvested for scRNAseq and/or IF.

### Quantification of γ-H2AX stained foci

Each well of 24-well plates was individually seeded with a single cell derived from either an epithelial-like (meroclone) or a mesenchymal-like (holoclone) cell. For immunofluorescence (IF) experiments cells were seeded onto coverslips in 24-wells plate. Post appropriate treatments, at the experimental end-points, cells were washed with PBS, fixed with 3% paraformaldehyde and permeabilized with 0.5% Triton X-100. Cells were blocked with 1% BSA for 1 h, then incubated sequentially with primary antibody (γ-H2AX, Millipore, 05–636) and secondary antibody (Alexa Fluor 488-conjugated goat anti-mouse antibody, Life Technologies, A21121) for 1 h each at 37°C, with three PBS washes in between. Coverslips were mounted onto glass slides with VECTASHIELD Mounting Medium with DAPI (Vector Labs, H-1200). Images were captured at 20× objective using a Nikon Eclipse TE2000-U microscope. Images of the same group were captured with identical exposure time. Images were processed using ImageJ software, and cells were scored as displaying either diffused or punctuated staining. Cells with punctuated staining were further analyzed for calculation of the number of foci. The experiment was performed at least thrice, and data analyzed using a two-tail *t*-test.

### Image cytometry of cell lines

For the assessment of single-cell variation in a population of cells Cell Profiler software (https://cellprofiler.org/) was used to extract quantitative feature information from images. TIFFs images were exported from IncuCyte ZOOM^TM^ to Cell Profiler software^106^. The Cell Profiler pipeline included: pre-processing, smoothing using smooth-keeping edges method, primary object identification from smoothed image using the global thresholding otsu method and all measurements describing state of every single cell, including primary object area, shape and intensity were exported. The measured features were then analyzed using dimension reduction methods to project the high dimensional data in 2D-plane. Single cells were scored by cellular radius, area, perimeter, solidity, formfactor, eccentricity and compactness to describe the morphological state and classify into holoclone-and meroclone-like morphologies. Two distinct classes of cells were identified based on cell morphology, termed E and M. E cells were flattened cells with irregular boundaries, more like epithelial-like cells comprising meroclone colonies. M cells were relatively small, circular and compact in shape, more like mesenchymal-like cells comprising holoclone colonies. Cell identities were then mapped onto X vs Y location dot plots called phenotype state maps.

### Scratch wound assay

Cells were grown to confluence in 24-well plates in IncuCyte ZOOM^TM^ system and the monolayer was scratched manually using a 10 µL Pipette tip. Plates were placed back in IncuCyte ZOOM^TM^ system and photographed every 2 hours for 48 hours to monitor wound closure as cells move to fill in the denuded region. Cell Profiler software was then used to extract quantitative feature information from images^106^. The Cell Profiler pipeline included: pre-processing, smoothing using smooth-keeping edges method, primary object identification from smoothed image using the global thresholding otsu method and all measurements describing state of every single cell, including primary object area, shape and intensity were exported. For wound closure measurement, in the Cell Profiler pipeline for image cytometry, we added MeasureImageAreaOccupied module.

### Clonogenic assay

Clonogenic assay was used to evaluate the proliferation of the holoclones and meroclones compared to the parental cells. The clonogenic assay is based on the growth of colonies from single cells, and IncuCyte ZOOM^TM^ system was used to detect colonies in label-free assay using time-lapse imaging. Colonies were inspected and scored on morphology and the size and number of cells per colony. The percentage of successful colonies observed in comparison with cells seeded (termed as clonal efficiency) was calculated by the following Eq. (1):

1 $${{{{{\rm{Clonal}}}}}}\,{{{{{\rm{efficiency}}}}}}=\frac{{{{{{\rm{number}}}}}}\,{{{{{\rm{of}}}}}}\,{{{{{\rm{colonies}}}}}}\,{{{{{\rm{observed}}}}}}}{{{{{{\rm{number}}}}}}\,{{{{{\rm{of}}}}}}\; {{{{{\rm{colonies}}}}}}\; {{{{{\rm{seeded}}}}}}}\times 100$$

The results were analyzed statistically using GraphPad Prism 5.01 software (GraphPad Software, Inc.).

### Single cell transcriptomic profiling of bladder cancer cell line

Monoclonally-derived holoclone and meroclone colonies of T24 cells, treated or untreated control, were trypsinized and resuspended in culture medium only (without FBS and antibiotics). Cell viability and numbers were determined using a cell counter, with viability <80% and numbers adjusted to 1×10^6^ cells/ml. Single cells were profiled using 10X single-cell RNAseq and Cell Ranger was used to generate expression matrix. Standard single-cell transcriptomic analysis was performed using Seurat^107^. Velocity analysis was performed using Velocyto software^108^. MAGIC^109^ algorithm was used for expression recovery or imputation which replaces zeros with imputed expression values for respective genes in dropout events of single‐cell transcriptomic data. For hallmark pathway analysis, hallmark genesets were used (http://www.broad.mit.edu/gsea/).

### Analysis of single-cell transcriptomic data of bladder tumors and cell lines

The single-cell RNA sequencing (scRNA-seq) data for UMUC3 cell line was obtained from the Gene Expression Omnibus (GEO) (GSE164041)^107^. The single cell transcriptomic dataset of patients with UBC were obtained from Chen et al. paper along with cell type annotations, which we refer to as the BLCA-SC dataset^10^. Standard single-cell transcriptomic analysis was performed using Seurat^107^ and only epithelial cells were used for further steps of Seurat SCTransform workflow, followed by principal component analysis (PCA), Uniform Manifold Approximation and Projection (UMAP) dimensionality reduction, clustering, pathway level activity scores, and subclonal inference to detect heterogeneity at genetic and non-genetic levels.

### Inference in clonal architecture

We used InferCNV (InferCNV of the Trinity CTAT Project. https://github.com/broadinstitute/inferCNV) package to estimate copy number status from single-cell transcriptomic data in tumors tissue samples, using normal tissue samples as reference. We used HMM-based prediction to determine subclonal events, which provides altered region coordinates, state assignments and clustering^30,57^. From the inferred copy number status, the cells were ordered by hierarchically clustering, major subclones were annotated and phylogeny tree was constructed. The copy number status was visualized in a heatmap illustrating the relative expression intensities across each chromosome with respect to the normal reference cells.

### Spatial transcriptomic profiling of bladder cancer tumors

Deidentified human tumor tissue samples subjected to spatial transcriptomic profiling were collected with written informed consent and ethics approval by the Rutgers Cancer Institute of New Jersey Institutional Review Board under protocol no. Pro2019002924 (PI: De). Briefly, 5 μm tissue sections were placed on the Visium Spatial Gene Expression Slide for FFPE following Visium Spatial Gene Expression for FFPE-Tissue Preparation Guide (10X Genomics, CG000408). Each slide had 4 capture areas and each capture area was 6.5 x 6.5 mm with ~5000 spots per capture area. Each spot was 55 µm in diameter with a 100 µm center to center distance between spots. Slides containing the tissue sections were incubated at 42 °C for 3 h and dried overnight at room temperature. Deparaffinization was then performed following Visium Spatial for FFPE – Deparaffinization, H&E Staining, Imaging & Decrosslinking Protocol (10X Genomics, CG000409). Slides were then used with Visium Spatial Gene Expression for FFPE User Guide (10X Genomics, CG000407) to generate Visium Spatial Gene Expression – FFPE libraries and sequenced on Illumina NovaSeq S4 300 cycle. The sequence data (FASTQ files) were processed using Space Ranger (v2.0.1) count pipeline for single-library analysis of fresh frozen (FF) and formalin-fixed paraffin embedded (FFPE) samples to align transcriptomic reads to the human reference genome (GRCh38), map them to the microscopic image of the tissue from which the reads were obtained and generate Feature Barcode matrices. The resulting count matrices and associated H&E physiological images were then used by the R package Seurat (v4.3.0) for standard spatial transcriptomic analysis using default Seurat parameters^107^. The four datasets were integrated using the Seurat SCTransform integration workflow, followed by principal component analysis (PCA), UMAP dimensionality reduction (UMAP) and clustering. Tissue/cell types of each cluster were inferred, and clusters were further refined by plotting clusters onto the associated histology images and identifying marker genes. For each spatial barcode, the gene signature activity scores were determined based on the enrichment of the target genes of SMAD3, KLF4 and PPARG (https://maayanlab.cloud/Harmonizome/gene_set/). We used Spotlight for deconvolution of cell types in each spatial location and represented as scatter pie plots^110^. We represented the spatial map of the tissues with a neighborhood graph, and then used spatial PCA (sPCA) after taking into consideration the graph structure and used regression with a spatial lag model (lagsarlm), as implemented in the spatialreg R package, to perform multivariate regression using a published approach^57^.

### NETACT

NETACT^111^ is a computational framework that constructs transcription factor transcription factor-based gene regulatory networks using transcription factor activity by integrating generic transcription factor-target relationships from literature-based databases against the backdrop of related gene expression data. Through gene grouping, topology optimization and examining the literature for catalog of known regulatory interactions involving cancer-related transcriptional master regulators, we can reduce the complex network to derive a closed, minimalistic core network.

### RACIPE algorithm

RACIPE is a mathematical modeling algorithm that allows an extensive exploration of the dynamical properties of a gene regulatory network^112^. Only the network topology is provided as an input to simulation framework, which is then modelled as a set of x ordinary differential equations (where x is the number of nodes in the gene regulatory network). The change in concentration of each node in the network depends on the production rate of the node, the effect of regulatory links incident on the node (modelled as a shifted Hill’s function) and the degradation rate of the node. Each parameter in the set of unknown parameters for ordinary differential equations (ODEs) is randomly sampled from a biologically relevant range. The sampling of parameters is done so as to ensure that it generates a representative ensemble of models for a specific circuit topology. The range for production rate varies from 1 to 100 while the range for the degradation rates varies from 0.1-1 (arbitrary units). The fold change parameter associated with each link is assumed to be in the range of 1 to 100-fold. The Hills coefficients sampled are assumed to vary from 1 to 6. After such sampling, the set of parameterized ODEs is solved to get different possible steady-state solutions. The set of ODEs can be multistable, i.e., multiple sets of steady-state concentrations satisfy the set of ODEs. The program samples 50000 different sets of parameters. For each parameter set, RACIPE chooses a random set of initial conditions (*n* = 100) for each node in the network and solves, using Euler’s method, with the set of coupled ODEs that represent interactions among the different nodes in a network. For each given parameter set and initial conditions, RACIPE reports the steady-state values for each of the nodes in the network. The steady-state values were then Z-normalized where the z-normalized expression value ${Z}_{i}$ is given by the following Eq. (2):

2 $${Z}_{i}=\frac{{E}_{i}-{E}_{{mean}}}{{E}_{{std}}}$$

where ${E}_{i}$ is expression level of a given node at *i*-th steady state solutions, and ${E}_{{mean}}$ and ${E}_{{std}}$ are the mean and standard deviation of the expression levels of a given node across all its steady state solutions, respectively. The perturbation analyses were done by performing RACIPE analysis on the system by either over expressing (OE) or down expressing (DE) the given node by 20-fold. The Z-score normalization of these perturbation data was done with respect to the control case.

### TCGA RNAseq data analysis

Gene expression data from 427 patients (408 Primary Tumor and 19 Solid Tissue Normal) with bladder cancer (TCGA-BLCA) was downloaded from Genomic Data Commons (GDC) using R package TCGAbiolinks (version 2.18.0). The gene expression data was normalized using Trimmed mean of M-values (TMM) followed by voom transformation using limma R package (version 3.46.0). Gene-level data was obtained by computing maximum normalized expression value for each transcript. Sample-level enrichment scores for gene signatures and cell-specific marker genes from gene expression profile were computed using GSVA R package (version 1.38.2). Cellular compositions were estimated by employing a marker-based approach by ranking enrichment scores of marker genes to make scoring independent of the gene expression units and represented in UMAP plots with dominant cell type. Tumor purity (proportion of cancer cells in a sample) was estimated from tumor gene expression profile, by calculating abundance scores for each spatial barcode score for infiltrating immune and stromal cells. To reduce high-dimensional transcriptomic data into low-dimensional representations and cluster patients, embedding of dataset into UMAP space was performed. Gene and pathway signatures were obtained from MSigDB^31^.

### Clinical and survival analysis

Data of 4 independent bladder cancer microarray datasets^113–115^ and Sanchez-Carbayo et al.^116^ was obtained from a meta-analysis study by Riester et al.^64^. Normalized gene expression profiles were analyzed for association with clinical variables like invasive phenotype. In the TCGA bladder cancer (TCGA-BLCA) cohort, for a gene-by-gene and gene signature survival analysis, the patients with above-median expression of the gene- or signature-of-interest were put in the up-regulated groups, and the remaining in the down-regulated group, and R packages survival (version 3.3-1) and survminer (version 0.4.9) were used. Hazard ratio and *p* value were determined using SurvExpress tool^117^.

### Statistics and Reproducibility

All statistical analyses were performed using R version 4.2.3 (https://www.r-project.org/) and GraphPad Prism 5.01 software. Asterisks indicate statistical significance wherein ***P < 0.001, **P < 0.01; *P < 0.05; ns, nonsignificant. Statistical tests and corresponding *p* values are listed for respective analyses. Data processing relied heavily on the Tidyverse version 2.0.0 R packages (https://www.tidyverse.org/).

### Reporting summary

Further information on research design is available in the Nature Portfolio Reporting Summary linked to this article.

### Supplementary information

Supplementary Information

Description of Additional Supplementary Files

Supplementary Data 1

Supplementary Data 2

Reporting Summary

## Data Availability

Genomic data for the project has been deposited to the Sequence Read Archive (BioProject: PRJNA1033657). The source data behind Figs 2c–e, 3j, 4f, 5a, c, d, and 6c–e can be found in Supplementary Data 1. All other data are available from the corresponding author on reasonable request.

## Code availability

In-house scripts used in this manuscript for graphs and analyses, which include data processing, downstream analysis, and the scripts used to generate figures are publicly available on Zenodo repository^118^ and at https://github.com/sjdlabgroup/BLCA-resources.
