# Supplementary material for: Transcriptional state dynamics lead to heterogeneity and adaptive tumor evolution in urothelial bladder carcinoma
Source: Commun Biol. 2023 Dec 21;6:1292. doi: 10.1038/s42003-023-05668-3 (PMC10739805; doi:10.1038/s42003-023-05668-3)
Supplement: Supplementary file 1 — Supplementary Information [file 42003_2023_5668_MOESM1_ESM.pdf]

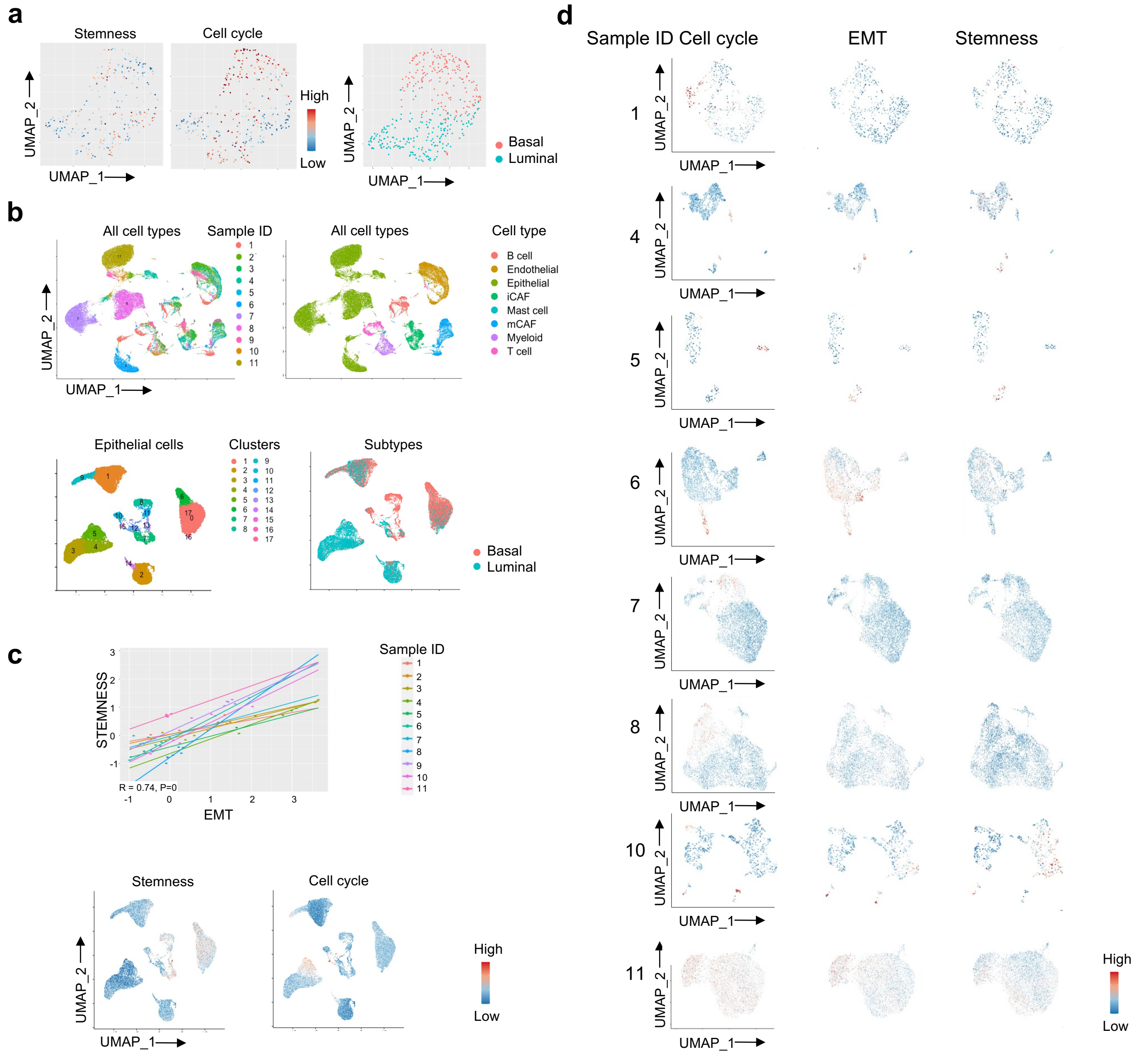

Supplementary Fig. 1: Characterization of transcriptional state clusters. a UMAP plots of TCGA bladder cancer (TCGA-BLCA) cohort of tumor only samples (n= 408) colored by oncogenic signatures. **b** UMAP plot of single cells of all cell types from BLCA-SC dataset colored by sample ID and cell types; and UMAP plots of epithelial single cells of all patient samples colored by cell clusters and subtypes. **c** Correlation analysis between oncogenic genesets where each dot is a subclonal entity from each tumor sample, and UMAP plots of epithelial single cells of all patient samples colored by oncogenic signatures. **d** UMAP plots of single cells of all tumor samples from BLCA-SC dataset colored by oncogenic phenotypes signatures. Each dot corresponds to a single cell and the color gradient is proportional to the expression of signature.

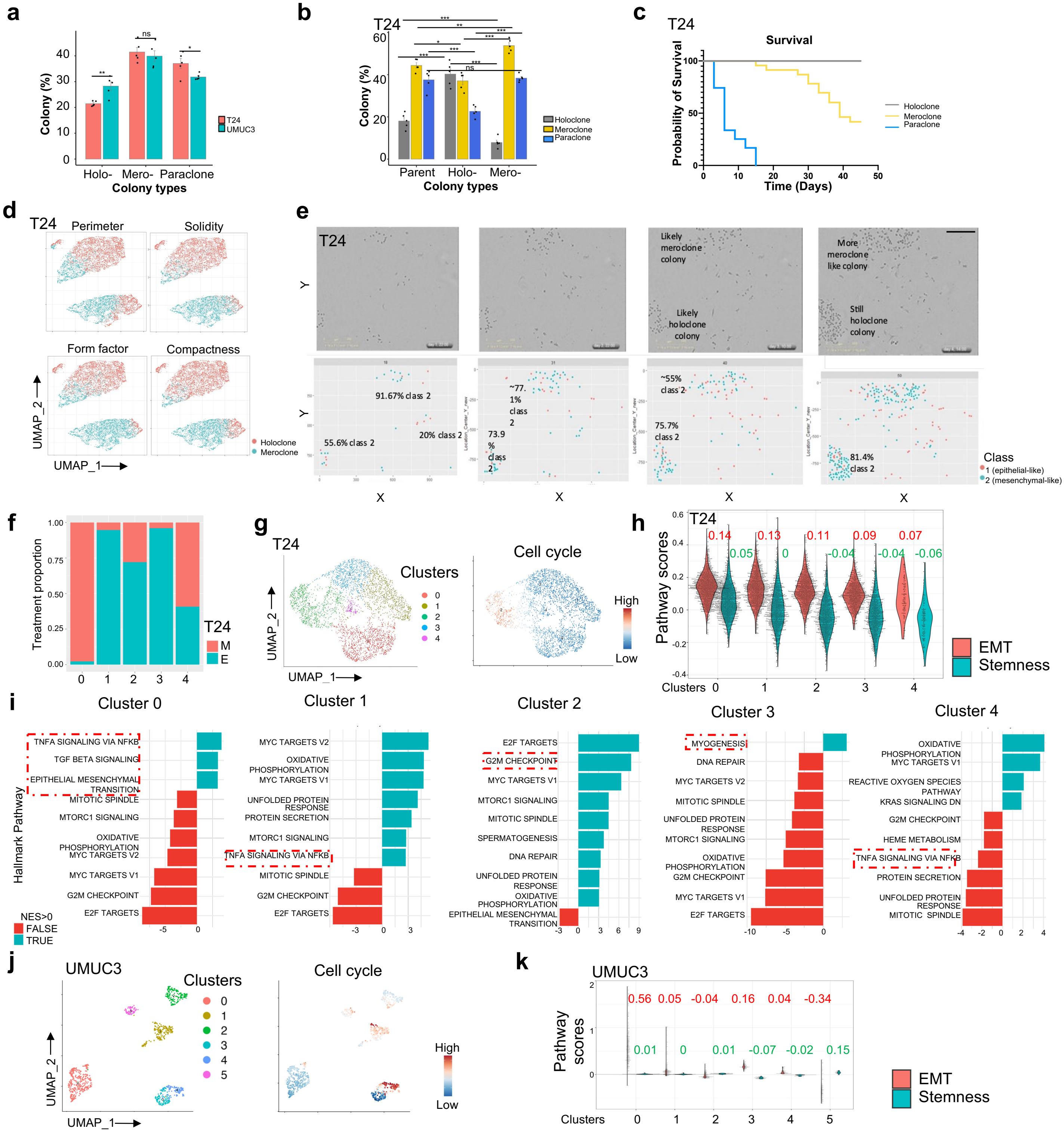

Supplementary Fig. 2: Bladder cancer cells exhibit unique transcriptional programs. **a** Quantitative analysis of different morphological colony types holo-, mero-, and paraclones derived from T24 and UMUC3 parental cells. Error bars represent standard deviation. **b** Self-renewal capacity of T24 parental cells and holo-, meroclones to form secondary colonies. Paraclone were unable to form secondary colonies. Error bars represent standard deviation. **c** Survival fate of holoclones (n=41), meroclones (n=46) and paraclones (n=31) from T24 cells during 45 days culturing and follow-up period. **d** UMAP plots of detected cellular objects colored by some of the cellular measurements used for identification of phenotypic classes in T24 cells. **e** Representative bright-field images and corresponding scatter plots post Cell profiler image segmentation and feature extraction of T24 cells using CellProfiler showing phenotypic classes Class 1 (epithelial-like) and Class 2 (mesenchymal-like) in phenotype states map. Scale bar represent 300 µm. **f** Stacked barplot showing composition of each cluster contributed by M and E phenotypes from scRNAseq data of T24 cells. **g** UMAP analysis showing the formation of main clusters in T24 derived M and E cells and annotated by cell cycle signature. **h** Violin plot representation of oncogenic signatures expression levels in T24 cells. **i** Results of GSEA Hallmark analysis for T24 scRNAseq data showing enriched signatures, with significant enrichment of Normalized Enrichment Score (NES) values for each cluster at FDR < 25% and p value < 0.05. Bar length represents the mean log2(fold change). **j** UMAP analysis showing the formation of main clusters scRNAseq data of UMUC3 cells and annotated by cell cycle signature. **k** Violin plot representation of oncogenic signatures expression levels in UMUC3 cells.

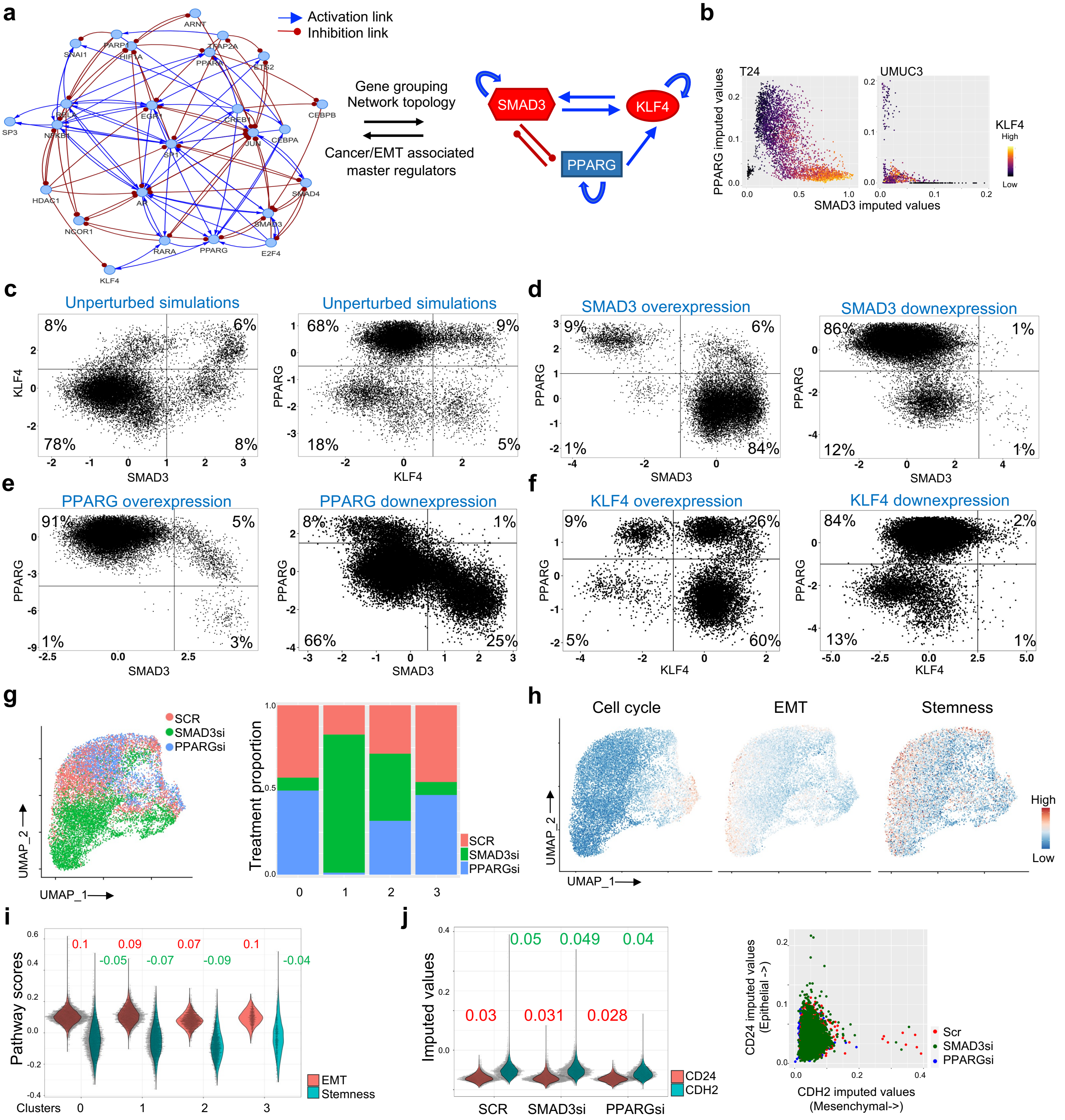

Supplementary Fig. 3: Effect of perturbations of regulatory network on gene expression variability and cell state. a Gene regulatory network and inferred minimalistic core network showing crosstalk between indicated genes. Blue lines and arrowheads represent the gene activation; red lines and blunt heads represent gene inhibition. **b** 2D scatter plot of imputed expression values of indicated genes in single cells at M and E transcriptomic states of T24 cell line and cells of UMUC3 cell line. **c-f** Scatter plot showing scores for all RACIPE solutions, and the biological phenotypes. **g** UMAP analysis showing siRNA treatment and stacked barplot showing composition of each cluster from scRNAseq data of gene expression perturbation in T24 cells, wherein SCR, SMAD3si and PPARGsi indicate scrambled control, SMAD3 and PPARG siRNAs respectively. **h** UMAP analysis showing cell clusters from siRNA treated cells, annotated by oncogenic signatures. **i** Violin plot representation of imputed expression values of indicated oncogenic pathways in different cell clusters from scRNAseq data of gene expression perturbation in T24 cells. **j** Violin plot and 2D scatter plot representation of imputed expression values of indicated genes in different treatment types from scRNAseq data of gene expression perturbation in T24 cells.

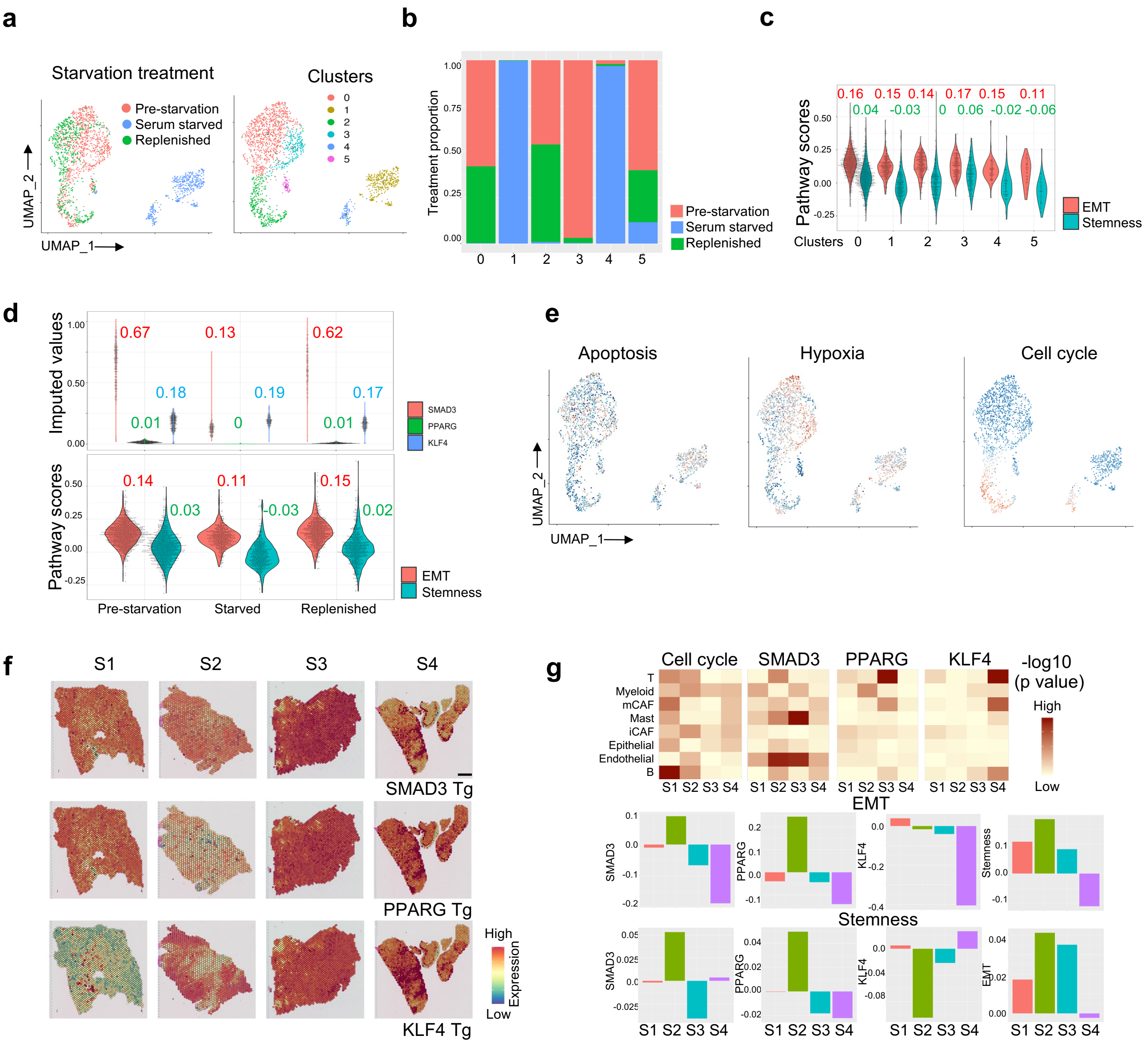

Supplementary Fig. 4: Transcriptional architecture of bladder tumor and its surrounding microenvironment. a UMAP plot of single cells showing the formation of main clusters and treatments in serum starvation experiment. **b** Stacked barplot showing composition of each clusters contributed by treatment types. **c-d** Violin plot representation of relevant genes and oncogenic signatures expression levels in different cell clusters (c) and treatment types (d) from pre-starvation, serum starved and replenished cells, with median values on top of plot. **e** UMAP analysis showing expression of indicated genes/pathway signatures. **f** Expression scores of target genes of SMAD3, PPARG and KLF4 from spatial transcriptomics data for the four bladder cancer specimens, indicated as S1-S4. Scale bars represent 1 mm. **g** Pathway or gene activity modeled as a function of the estimated abundance of the cell types or function of expression of relevant genes and pathway using multivariate regression with a spatial lag to account for spatial autocorrelation, with heatmap showing p-value associated with coefficients for the cell types and barplots showing coefficients from spatial transcriptomics data for the four bladder cancer specimens, indicated as S1-S4.

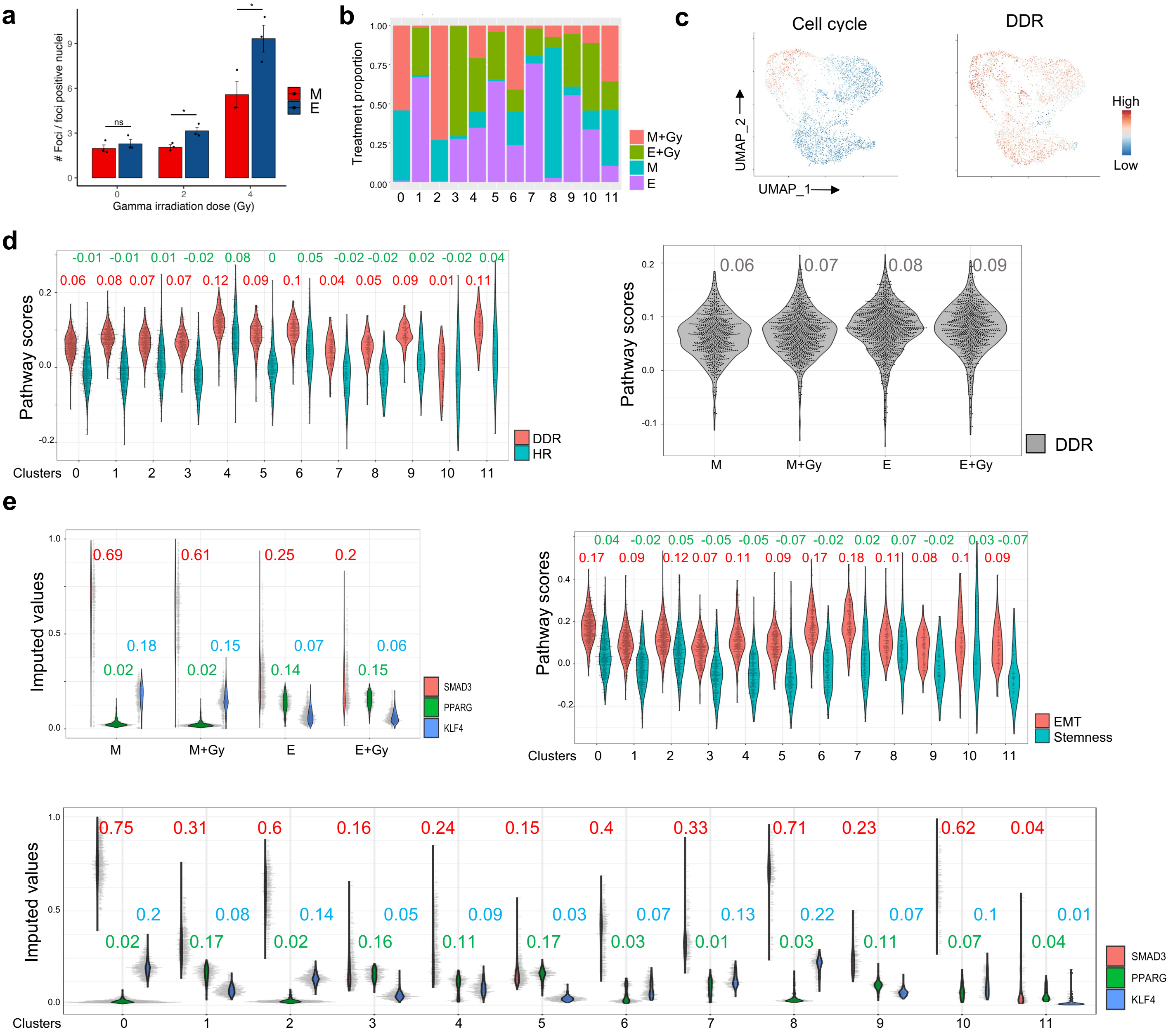

Supplementary Fig. 5: Bladder cell lines exposed to radiation perturbations. a Quantification of γ-h2ax foci upon irradiation in T24 cells with error bars representing SD. P-value was calculated using t-test wherein *p-value < 0.05. **b** Stacked barplot showing composition of each cluster contributed by treatment type. UMAP plots showing cell cycle phase of single cells and expression cell cycle signature **c-e** UMAP analysis and violin plot quantification showing cell clusters/treatments to view expression of indicated genes and pathway signatures with median value on top of violin plot.

a

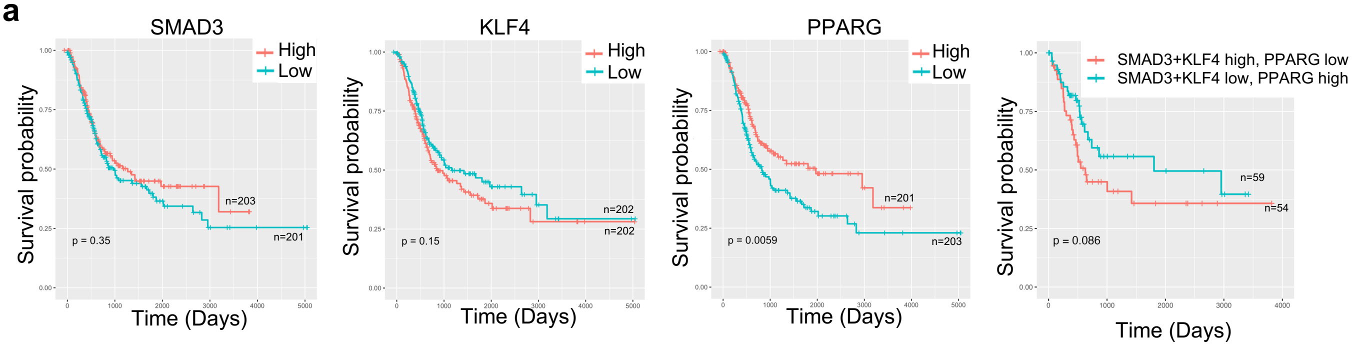

b **c**

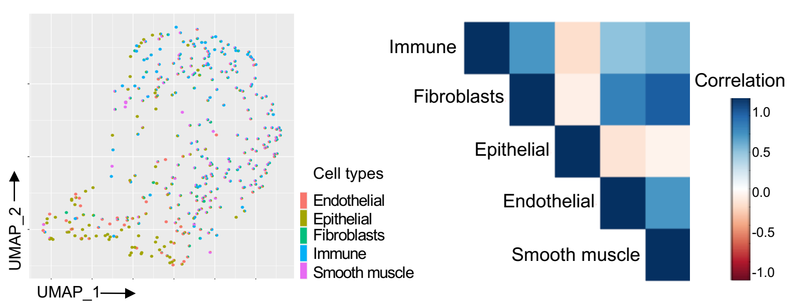

d

Cell type	N	Hazard ratio (95% CI)	P value
Immune	N=408	0.67 (0.47-0.96)	0.028
Fibroblasts	N=408	1.88 (0.96-3.66)	0.064
Epithelial	N=408	1.16 (0.68-1.98)	0.591
Endothelial	N=408	1.11 (0.76-1.62)	0.601
Smooth muscle	N=408	0.96 (0.59-1.56)	0.867

Supplementary Fig. 6: Association between cell states and prognosis. **a** Overall survival analysis of TCGA-BLCA cohort according to differential expression (median) of indicated genes by Kaplan-Meier analysis. Red line represents high gene expression; blue line, low expression. p-values were calculated using Log-rank test. **b** UMAP plot displaying inferred dominant cell type of each sample from TCGA-BLCA cohort using deconvolution. **c** Correlation matrix (pearson correlation) of abundance scores of major cell types in the TCGA-BLCA tumors. Abundance scores of major cell types were estimated using deconvolution of bulk RNAseq data for each sample. **d** Hazard ratio of each sample from TCGA-BLCA cohort annotated by dominant cell type. p-values were calculated using Log-rank test.
