# Supplementary material for: Transcriptional state dynamics lead to heterogeneity and adaptive tumor evolution in urothelial bladder carcinoma
Source: Commun Biol. 2023 Dec 21;6:1292. doi: 10.1038/s42003-023-05668-3 (PMC10739805; doi:10.1038/s42003-023-05668-3)
Supplement: Supplementary file 2 — Description of Additional Supplementary Files [file 42003_2023_5668_MOESM2_ESM.pdf]

Description of Additional Supplementary Files

File name: Supplementary Data 1
Description: Source data for Fig 2c-e, 3j, 4f, 5a, 5c-d, and 6c-e.

File name: Supplementary Data 2
Description: Genes defining epithelial-mesenchymal transition, as in wound healing, fibrosis and metastasis.
